# A Screening for Virus Infections in Eight Herds of Semi-domesticated Eurasian Tundra Reindeer (*Rangifer tarandus tarandus*) in Norway, 2013–2018

**DOI:** 10.3389/fvets.2021.707787

**Published:** 2021-10-12

**Authors:** Morten Tryland, Javier Sánchez Romano, Ingebjørg Helena Nymo, Eva Marie Breines, Francisco Javier Ancin Murguzur, Ole Christian Kjenstad, Hong Li, Cristina W. Cunha

**Affiliations:** ^1^Department of Arctic and Marine Biology, UiT the Arctic University of Norway, Tromsø, Norway; ^2^Department of Forestry and Wildlife Management, Inland Norway University of Applied Sciences, Koppang, Norway; ^3^Norwegian Veterinary Institute, Tromsø, Norway; ^4^Animal Disease Research Unit, Agricultural Research Service, US Department of Agriculture, Pullman, WA, United States; ^5^Department of Veterinary Microbiology and Pathology, College of Veterinary Medicine, Washington State University, Pullman, WA, United States

**Keywords:** alphaherpesvirus, bluetongue virus, gammaherpesvirus, parapoxvirus, pestivirus, schmallenberg virus, serology

## Abstract

**Background:** Previous serological screenings have indicated that Eurasian semi-domesticated tundra reindeer (*Rangifer tarandus tarandus*) in Finnmark, Northern Norway, are exposed to alphaherpesvirus, gammaherpesvirus and pestivirus. Alphaherpesvirus (i.e., Cervid herpesvirus 2; CvHV2) has been identified as the transmissible component of infectious keratoconjunctivitis (IKC). Limited knowledge exists on the presence and prevalence of virus infections in other herding regions in Norway, which are hosting ~67,000 semi-domesticated reindeer and have contact with other species and populations of wildlife and livestock than those present in Finnmark.

**Methods:** Blood samples (*n* = 618) were obtained over five winter seasons (2013–2018), from eight different herds representing summer pasture districts in Tana, Lakselv, Tromsø, Lødingen, Hattfjelldal, Fosen, Røros, and Filefjell, distributed from North to South of the reindeer herding regions in Norway. Blood samples were investigated for specific antibodies against five viral pathogen groups, alphaherpesvirus, gammaherpesvirus (viruses in the malignant catarrhal fever group; MCFV), pestivirus, bluetongue virus (BTV), and Schmallenberg virus (SBV), by using commercial multispecies serological tests (ELISA). In addition, swab samples obtained from the nasal mucosal membrane from 486 reindeer were investigated by PCR for parapoxvirus-specific DNA.

**Results:** Antibodies against aphaherpesvirus and MCFV were found in all eight herds, with a total prevalence of 42% (range 21–62%) and 11% (range 2–15%), respectively. Anti-Pestivirus antibodies were detected in five of eight herds, with a total prevalence of 19% (range 0–52%), with two of the herds having a particularly high seroprevalence. Antibodies against BTV or SBV were not detected in any of the animals. Parapoxvirus-specific DNA was detected in two animals representing two different herds in Finnmark.

**Conclusions:** This study confirmed that alphaherpesvirus and MCFV are enzootic throughout the geographical reindeer herding regions in Norway, and that pestivirus is present in most of the herds, with varying seroprevalence. No exposure to BTV and SBV was evident. This study also indicated that semi-domesticated reindeer in Finnmark are exposed to parapoxvirus without disease outbreaks being reported from this region.

## Introduction

Reindeer husbandry is of major importance for livelihoods and for cultural values in Norway. For the five winter seasons of 2013–2018 (this study), the total number (all numbers being mean over five winters, counted March 31st, after slaughter and before calving) of Eurasian tundra reindeer (*Rangifer tarandus tarandus*) was reported to be 217,824 animals, with 74,441 animals slaughtered per year, representing 1,689 tons of meat. In Norway, reindeer herding is for the most part conducted by Sami herders, except for a few herds in the southernmost part of the reindeer herding region. In most areas in the country, reindeer herding is characterized as a mountain tundra type, with intermediate to long seasonal animal migration, using inland tundra pastures during winter. While in a few regions a coastal-oriented type is conducted, with local seasonal migrations, using coastal and snow free areas during winter (1). In many regions, reindeer share pasture with sheep, and to some extent goats. In addition, they may have contact with wild ruminants, such as moose (*Alces alces*), roe deer (*Capriolus capriolus*), and in some regions red deer (*Cervus elaphus*). A shortening daylength during fall increase melatonin levels in reindeer, which is associated with onset of puberty in young animals, oestrus in females, and rutting in males (2). Reindeer normally have a single offspring, born in spring. Reindeer are gregarious animals, forming large herds. The production is for the most part based on natural pastures, but the practice of supplementary feeding is increasing and may contribute to increased animal-to-animal contact and poor hygiene on feeding spots which may facilitate transmission of potential pathogens (3).

During 2013–2018 (this study), the average annual animal losses were reported to be 60,925 calves and 21,877 adult reindeer. The majority of these losses was reported to be due to predators, being on average for the period 89% of the lost calves and 77% of the lost adult reindeer. Loss of animals with unknown cause was reported to be 8% for calves and 13% for adult reindeer (4).

There have been no reports of major mortalities due to specific infectious diseases during the five winter seasons covered by this study. Previous serological screenings of semi-domesticated reindeer indicated that reindeer alphaherpesvirus, gammaherpesvirus (malignant catarrhal fever virus group; MCFV), and pestivirus are enzootic in all the investigated herds of Finnmark, Northern Norway (5–10), hosting ~73% of all semi-domesticated reindeer in Norway.

The alphaherpesvirus circulating in reindeer in Fennoscandia has been identified as Cervid herpesvirus 2; CvHV2, which is grouped with other virus species in the subfamily *Alphaherpesvirinae*, genus *Varicellovirus*, that infect cattle, goats, and cervids. Serological screenings have indicated that CvHV2, or similar alphaherpesviruses, are enzootic in most of the investigated reindeer and caribou populations, including Sweden, Finland, Alaska (USA) and Canada (11–13). A serosurvey conducted among semi-domesticated reindeer in Finnmark, Norway (2004–2006, *n* = 3,062), showed that seroprevalence increased with age, being 8% in calves (≤1 year) and 49% in adults (>1 year) (7). It has been shown that IKC may cause clinical symptoms characteristic for infectious keratoconjunctivitis (IKC) in semi-domesticated reindeer (14), often associated with secondary bacterial infections (15). IKC may affect single animals in a herd, but may also appear as regular outbreaks, affecting animal welfare and causing mortality, especially in young and immunologically naïve animals (16).

The disease malignant catarrhal fever (MCF) is caused by MCFV, a group of gammaherpesviruses that primarily affect domestic and wild ruminants (17, 18). Antibodies against MCFV were previously found in semi-domesticated reindeer (*n* = 3,339) in Finnmark, with a prevalence of 3.5% (8). The risk of exposure was significantly higher for adults (>1 year) than for calves (≤1 year), higher in the eastern compared to the western part of the studied area and increased with increasing population density (8). Further, a clinical case of sheep-associated MCF has been 208 diagnosed in a semi-domesticated reindeer (16). One clinical 209 reindeer case of malignant catarrhal fever has been reported (16), 210 but the overall impact of such infections in the reindeer herds is 211 not known.

*Pestivirus* is a genus in the *Flaviviridae* family, with four well-defined species causing disease in livestock; Pestivirus A and B (former BVDV-1 and BVDV-2, respectively) associated with cattle, Pestivirus C in swine (former classical swine fever virus; CSFV) and Pestivirus D in sheep (former border disease virus; BDV). In a study of 48 carcasses of predominantly emaciated semi-domesticated reindeer from Finnmark, pestivirus antibodies were detected in 33% of the animals by using a virus neutralization test (VNT) (6). A larger screening in Finnmark of apparently healthy reindeer sent to slaughter (2004–2008, *n* = 3,339) revealed a prevalence of 13% (9). In Sweden, several studies have shown a seroprevalence ranging from 0 to 35% among different reindeer herds (19, 20). A recent screening (2016–2017) revealed a mean seroprevalence of 49% in three Swedish herds and 41% in three Norwegian herds (21). In wild Eurasian tundra reindeer (*R. t. tarandus*) in southern Norway, a seroprevalence of 4.2% was found (22), whereas a seroprevalence ranging from 34 to 78% has been described in different subspecies (*R. t. granti, R. t. caribou*, and *R. t. groenlandicus*) and populations of migratory caribou herds across Canada and in western Greenland (13).

Parapoxvirus (family *Poxviridae*) may cause proliferative processes in the skin and oral mucosa of reindeer, a disease called contagious ecthyma (CE) (16). Contagious ecthyma has been reported in semi-domesticated reindeer in Sweden (23), Norway [experimental animals; (24)] and Finland, the latter country having experienced severe outbreaks affecting tens and hundreds of animals, sometimes with high mortality (25). The single reported serious outbreak of CE under regular reindeer herding conditions in Norway appeared in Nordland in April 2000. It was caused by Orf virus (ORFV) and affected about 30 animals (26). Since reindeer's humoral immune response against parapoxvirus is assumed to be short lived, as in sheep (27), serological tests may be of restricted value. Active infections may be detected by the amplification (polymerase chain reaction; PCR) of parapoxvirus-specific DNA in tissue or swab samples, and phylogeny studies based on amplicon DNA sequences may help characterizing the circulating virus (28).

Bluetongue virus (BTV; genus *Orbivirus*, family *Reoviridae*) is transmitted by biting midges (*Culicoides* spp.) and may cause non-contagious acute disease in naïve sheep, inducing fever, excessive salivation, and oedema and cyanosis of the tongue and lips. BTV also infects cattle and a wide range of other domestic animals. Wild cervids are regarded as important in the epidemiology of BTV (29). Due to a rapid northward distribution of BTV in Europe, BTV appeared in Denmark (2007), Sweden (2008), and Norway (2009), but exposure of reindeer has not been documented (16). However, antibodies against BTV were detected in captive reindeer in Germany, with a prevalence of 3.4% (*n* = 119) (30).

Schmallenberg virus (SBV; genus *Orthobunyavirus*, family *Bunyaviridae*) appeared in dairy cattle in Germany in 2011, quickly spreading to 27 European countries during in the following couple of years (31, 32), including Norway (33). As for BTV, no exposure of semi-domesticated or wild reindeer has been reported for SBV, but antibodies were detected in captive reindeer in Germany, with a prevalence of 59% (*n* = 115) (30), indicating that reindeer are susceptible to infection.

The virus infections mentioned above were selected for this study since there are evidence that reindeer are exposed to these viruses, showing that reindeer are susceptible to infection. Alpha- and gammaherpesviruses and parapoxviruses may cause specific diseases in reindeer, whereas such knowledge is scarce or absent for pestivirus, BTV and SBV. However, comparable studies and reports on domestic animals and wild ruminants suggest that also reindeer may be clinically affected upon exposure. Except for the region of Finnmark, little is known about the exposure of semi-domesticated reindeer to viral pathogens in the other reindeer herding regions in Norway, hosting about 67,000 animals. The aim of this study was to investigate the exposure of semi-domesticated reindeer to six viral pathogens in eight different herding districts, representing a geographical north to south gradient through the reindeer herding regions of Norway and, at the same time, addressing possible temporal trends during the sampling period of five winter seasons.

## Materials and Methods

### Animals and Sampling

**Figure 1:**
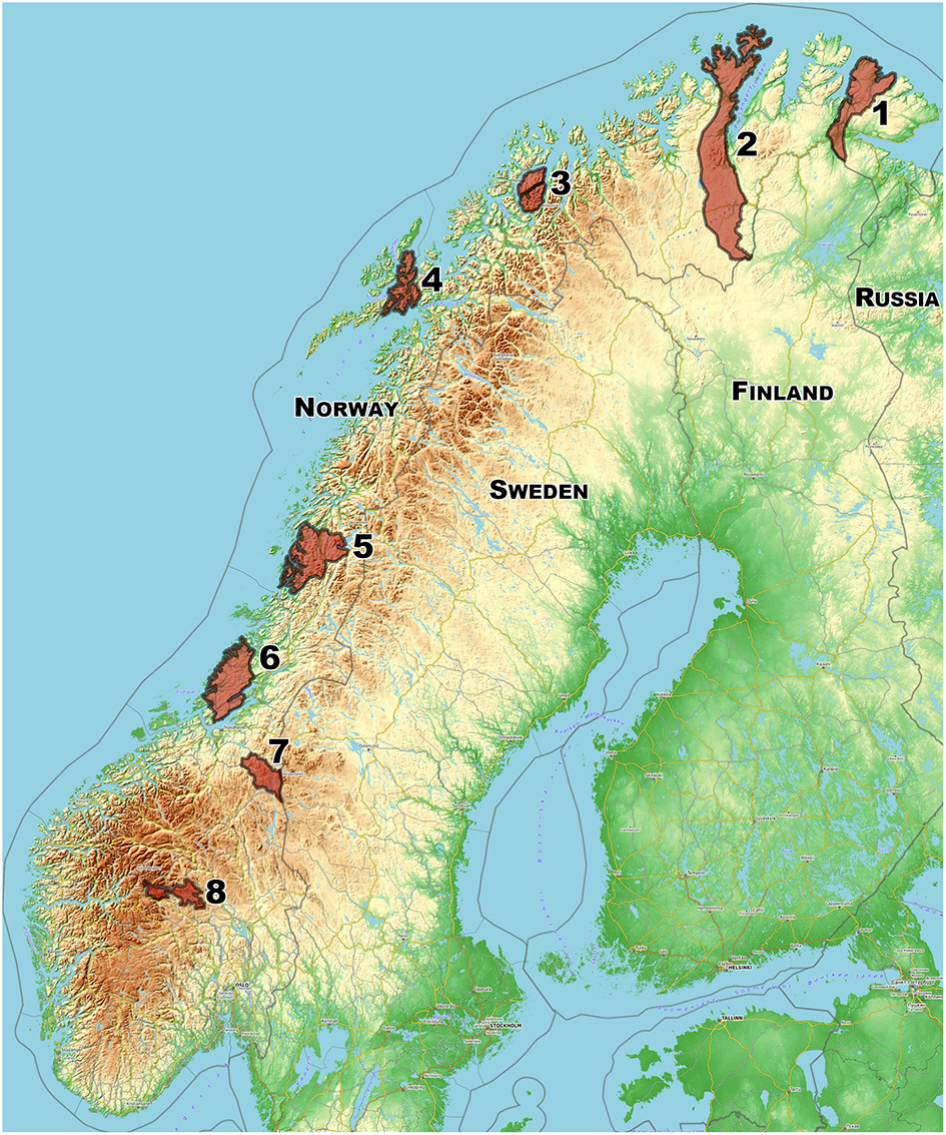
Map showing the approximate geographical positions for the sampling of eight Norwegian herds of semi-domesticated Eurasian tundra reindeer (*Rangifer tarandus tarandus*), distributed from south to north of the reindeer herding regions of Norway and screened for selected viral infections (*n* = 618; 2013–2018). Map created using the Free and Open Source QGIS. Map data: ©OpenStreetMap-Mitwirkende, SRTM | Map position: ©OpenTopoMap (CC-BY-SA).

Eight different semi-domesticated reindeer herds were sampled during late fall and winter (October - April) in five consecutive winter seasons, during the period 2013–2018. Whenever possible, samples were obtained from 10 calves (4–10 months old; both sexes) and 10 adult females (>1 year) from each of the eight herds (Figure 1). A total of 618 animals were sampled, 294 adults and 324 calves of the year (Table 1). The animals were either sampled live and released back into the herd during routinely herding practices or sampled before or during the slaughter process. Blood samples were collected from the jugular vein in blood tubes (BD Vacutainer® BD, Plymouth, UK), using a venoject needle (Terumo, Leuven, Belgium) for live animals, or by collecting blood directly into open tubes during bleeding of slaughtered animals. Serum was prepared by centrifugation (10 min, 3.000 *g*) and stored at −20°C until further analyses. Swab samples (Applimed SA, Châtel-St-Denis, Switzerland) were obtained from 486 reindeer by gently rubbing the mucosal membrane of the nose ~5 cm inside one of the nostrils. Swabs were placed in 1.8 ml cryotubes with 800 μl of Eagle's Minimum Essential Medium (EMEM) containing antibiotics (10,000 U/ml penicillin and 10 mg/ml streptomycin; 1 ml/l of gentamicin 50 mg/ml and 10 ml/l of amphotericin B 250 μg/ml; EMEMab 10 ml/l), frozen and stored at −80°C until analysis.

**Table 1:** Serological investigation of semi-domesticated Eurasian tundra reindeer (*Rangifer t. tarandus*) from eight different reindeer summer pasture districts in Norway, 2013–2017, presented as seropositive/animals tested, and seroprevalence (%) with confidence interval in square brackets for females (F), males (M), and overall (O).

**Geographical region^a^**	**Sex**	**Alphaherpesvirus**	**Gammaherpesvirus**	**Pestivirus**
1. Tana	F	61/88; 69% (59;78)	4/88; 5% (2;11)	11/88; 13% (7;21)
	M	1/11; 9% (2;38)	0/11; 0 % (0;11)	3/11; 27% (10;57)
	O	62/99; 62% (53;72)	4/99; 4% (2;10)	14/99; 14% (9;22)
2. Lakselv	F	28/46; 61% (46;74)	7/46; 15% (8;28)	0/46; 0 % (0;7)
	M	4/13; 31% (13;58)	1/13; 7% (1;33)	0/13; 0 % (0;23)
	O	32/59; 54% (42;66)	8/59; 14% (7;24)	0/59; 0 % (0;6)
3. Tromsø	F	12/34; 35% (21;52)	1/35; 3% (1;15)	6/34; 18% (8;34)
	M	2/33; 6% (2;20)	3/35; 9% (3;22)	0/33; 0 % (0;10)
	O	14/67; 21% (13;32)	4/70; 6% (2;14)	6/67; 9% (4;18)
4. Lødingen	F	10/17; 59% (36;78)	1/17; 6% (1;27)	3/17; 17% (6;41)
	M	1/13; 8% (1;33)	0/13; 0 % (0;22)	0/13; 0 % (0,22)
	O	12^b^/42^b^; 29% (17;44)	1^b^/42^b^; 2% (0;12)	3^b^/42^b^; 7% (2;19)
5. Hattfjelldal	F	25/64; 39% (28;51)	9/51; 18% (10;30)	35/64; 55% (43;66)
	M	10/46; 22% (12;36)	4/38; 11% (4;24)	22/46; 48% (34;62)
	O	35/104; 34% (25;43)	13/89; 15% (9;23)	57/110; 52% (43;61)
6. Fosen	F	19/36; 53% (37;68)	7/34; 21% (10;37)	0/36; 0 % (0;10)
	M	7/24; 29% (15;49)	1/24; 4% (1;20)	0/24; 0 % (0;14)
	O	26/60; 43% (32;56)	8/58; 14% (7;25)	0/60; 0 % (0;6)
7. Røros	F	24/57; 42% (30;55)	9/57; 16% (9;27)	24/57; 42% (30;55)
	M	7/25; 28% (14;48)	3/25; 12% (4;30)	6/25; 24% (11;43)
	O	31/82; 38% (28;49)	12/82; 15% (8;24)	30/82; 37% (27;47)
8. Filefjell	F	11/31; 36% (21;53)	5/31; 16% (7;33)	0/31; 0 % (0;11)
	M	18/32; 56% (39;72)	3/32; 9% (3;24)	0/32; 0 % (0;11)
	O	29/65^b^; 45% (33;57)	8/65^b^; 12% (6;22)	0/65^b^; 0 % (0;5)
Overall	F	190/373; 51% (46;56)	43/359; 12% (9;16)	79/373; 21% (17;26)
	M	50/197; 25% (20;32)	15/191; 8% (5;13)	31/197; 16% (11,21)
	O	240/570^2^; 42% (38;46)	58/550^2^; 11% (8;13)	110/570^2^; 19% (16;23)

a *Geographical Regions (Counties): 1. Tana (Troms and Finnmark), 2. Lakselv (Troms and Finnmark), 3. Tromsø (Troms and Finnmark), 4. Lødingen (Nordland), 5. Hattfjelldal (Nordland), 6. Fosen (Trøndelag), 7. Røros (Trøndelag), 8. Filefjell (Innlandet)*.

b *Of the 42 animals from Lødingen, 12 calves were sampled with no record of sex, of which one was seropositive for alphaherpesvirus. None of the 12 calves had antibodies against any of the other pathogens tested. Similarly, of the 65 animals sampled in Filefjell, two were sampled with no record of sex, and being seronegative for all tests*.

### Analyses

A total of 618 reindeer were sampled for blood. The number of animals tested for antibodies against each pathogen, however, varied somewhat due to the limited volume of samples for some of the animals.

### Alphaherpesvirus

Serum samples (*n* = 570) were investigated for anti-alphaherpesvirus antibodies using a commercial bovine enzyme-linked immunosorbent assay (ELISA) based on BoHV1 glycoprotein B (gB) as antigen (Table 2). The kit has previously been validated against a virus neutralization test (VNT) for analyzing reindeer serum samples for anti-alphaherpesvirus antibodies (34). All serum samples were tested in duplicate and evaluated against bovine (provided with the kit) and reindeer (35) positive-control sera.

**Table 2:** Serological tests used to investigate semi-domesticated Eurasian tundra reindeer (*Rangifer t. tarandus*) from eight reindeer herding districts in Norway (2013–2015) for antibodies against viral pathogens.

**Viral pathogen**	**Test and producer**	**Test principle**	**Antigen**	**References**
Alphaherpes	SERELISA BHV-1 gB Synbiotics, France	Blocking ELISA	Glycoprotein B	(34)
MCFV	Non-commercial	Competitive-inhibition ELISA	AlHV-1^*^	(36)
Pesti	SERELISA® BVD p80 Synbiotics, France	Blocking ELISA	p80	(37)
Bluetongue	ID Screen® Bluetongue Competition. ID Vet, Grabels, France	Competitive ELISA	Recombinant, VP7	(38)
Schmallenberg	ID Screen® Schmallenberg virus competition multispecies. ID Vet, Grabels, France	Indirect ELISA	Recombinant SBV antigen	(39)

* *The MCFV cELISA is based on a monoclonal antibody (15-A) against an epitope highly conserved among all viruses in the MCF group, including AlHV1, AlHV2, OvHV2, CpHV2, CpHV-3, and Hippotragine herpesvirus 1 (HiHV1)*.

### MCFV

Serum samples (*n* = 550) were investigated for the presence of specific antibodies against viruses from the MCFV-group by a direct competitive-inhibition ELISA (cELISA) (36, 40), applied for reindeer serum samples as described previously (8) (Table 2). The cELISA is based on a monoclonal antibody (15-A) against an epitope highly conserved among all identified MCFV, including OvHV-2, CpHV-2, and CpHV-3 that are known also to infect and cause disease in cervids. Sera were tested as described for a previous screening of reindeer serum samples in Finnmark (8).

### Pestivirus

Serum samples (*n* = 570) were tested for anti-pestivirus antibodies using a commercial blocking ELISA (bELISA; Table 2) designed for domestic ruminants which is based on the p80/125 non-structural protein as antigen, presumably shared between all strains of Pestivirus A and B (BVDV) and D (BDV) (37). This kit has previously been used for testing red deer (*Cervus elaphus*) and other wild ruminants for pestivirus antibodies, including reindeer (9, 20, 22). Further details, including evaluation of the kit for testing reindeer serum samples by a virus neutralization test (VNT), is published elsewhere (9).

### Bluetongue Virus

Serum samples (*n* = 479) were investigated for antibodies against BTV using a commercial competitive ELISA based on recombinant VP7 as antigen (Table 2), which is conserved among BTV serotypes. According to the producer, the kit is suitable for testing multiple species, including sheep, goat, buffalo, deer, and others (38, 41), and has also been used to test reindeer serum samples (30). Sera were tested as described for a previous screening of reindeer serum samples (30).

### Schmallenberg Virus

Serum samples (*n* = 470) were investigated for antibodies against SBV using a commercial competitive ELISA based on recombinant SBV nucleoprotein antigens for multispecies testing (Table 2). This kit has been used to detect anti-SBV antibodies in red deer (Cervus elaphus), fallow deer (Dama dama), moose (Alces alces), and roe deer (Capreolus capreolus) (42), as well as reindeer (30). Sera were tested as described for a previous screening of reindeer serum samples (30).

### Parapoxvirus

DNA was isolated from nasal swab samples with a Maxwell 16 Buccal Swab LEV DNA purification kit (Promega, Madison, WI, USA). PCRs targeting the *B2L* gene (43) and the parapoxvirus gene region encoding a protein inhibiting granulocyte-macrophage colony-stimulating factor and interleukin-2 (*GIF*) (44) were conducted (28). Isolated DNA from a skin lesion of a goat with CE (Norwegian Veterinary Institute) was used as a positive control. PCR amplicons were prepared by enzymatic removal of unused dNTP and primers (ExoSAP-IT™; Amersham Pharmacia Biotech, Sweden) and subsequently sequenced (BigDye®Terminator v3.1 cycle sequencing kit; Applied Biosystems, Norway) in an Applied Biosystems 3130 XL Genetic Analyzer (Applied Biosystems).

### Statistical Analyses

All analyses were performed using the *glm* function in R 4.1.0 (45). The antibody screening for each virus infection was individually assessed with a binomial outcome (negative/positive result in the test, coded as 0 or 1, respectively) and a logit link, after removing rows with missing data in the explanatory variables. Explanatory variables comprised each individual's age (coded as calf or adult), sex (male or female), latitude, and sampling season (year). Age and sex were categorical variables, and latitude and sampling season were numerical variables. Goodness of fit and overdispersion were checked during the modeling, and the significance level was set at *P* < 0.05 for the explanatory variables.

## Results

**Figure 2:**
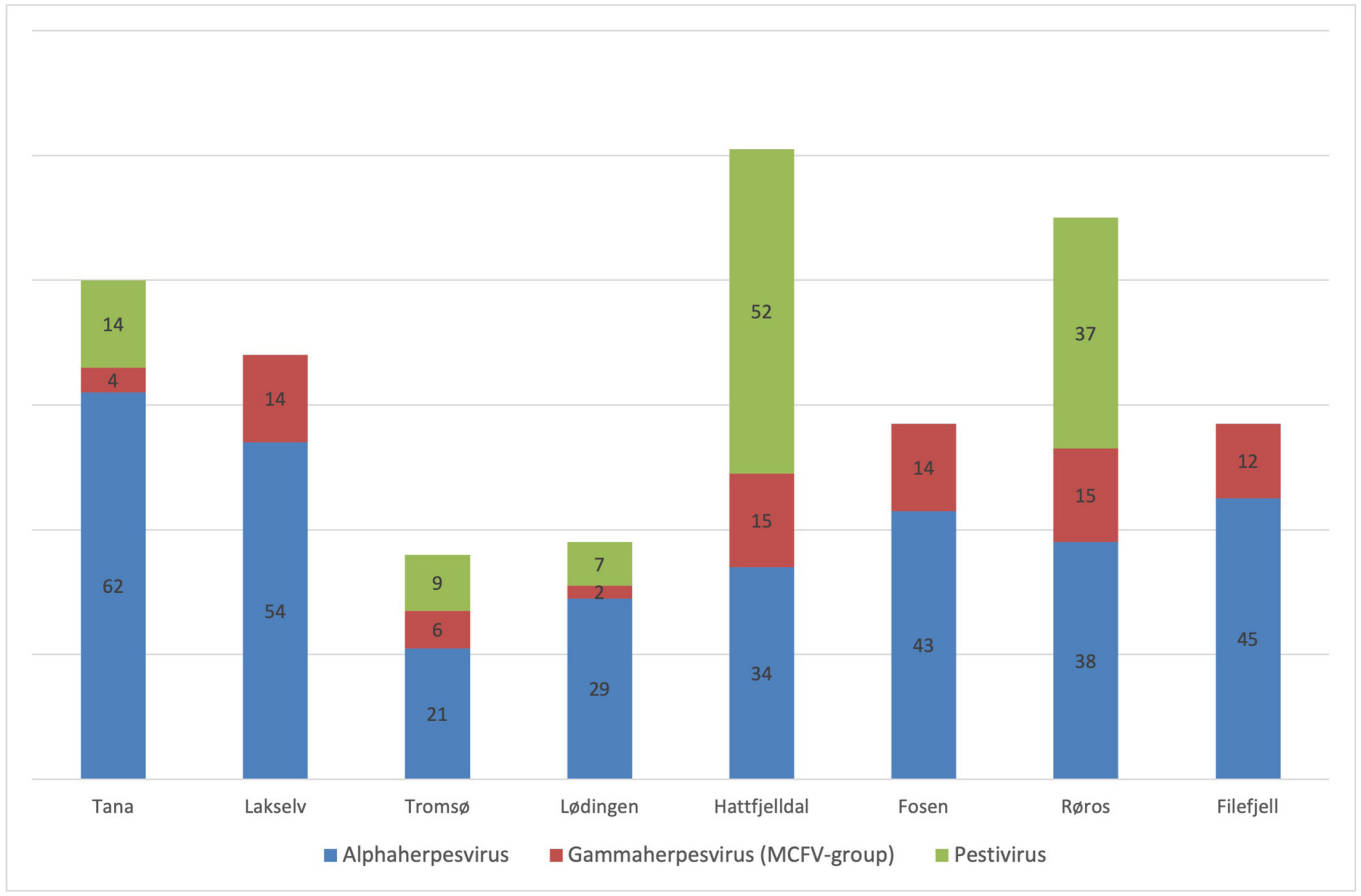
Seroprevalence (% on bars) of Alphaherpesvirus, Gammaherpesvirus (MCFV-group) and Pestivirus in semi-domesticated Eurasian tundra reindeer from eight geographical regions in Norway over five consecutive winter seasons (2013–2018).

Results from the serological screening for antibodies against alphaherpesvirus, MCFV and pestivirus are displayed in Table 1 and Figure 2. In Table 1, prevalence values are presented as a percentage with 95% confidence interval (CI) in square brackets.

**Figure 3:**
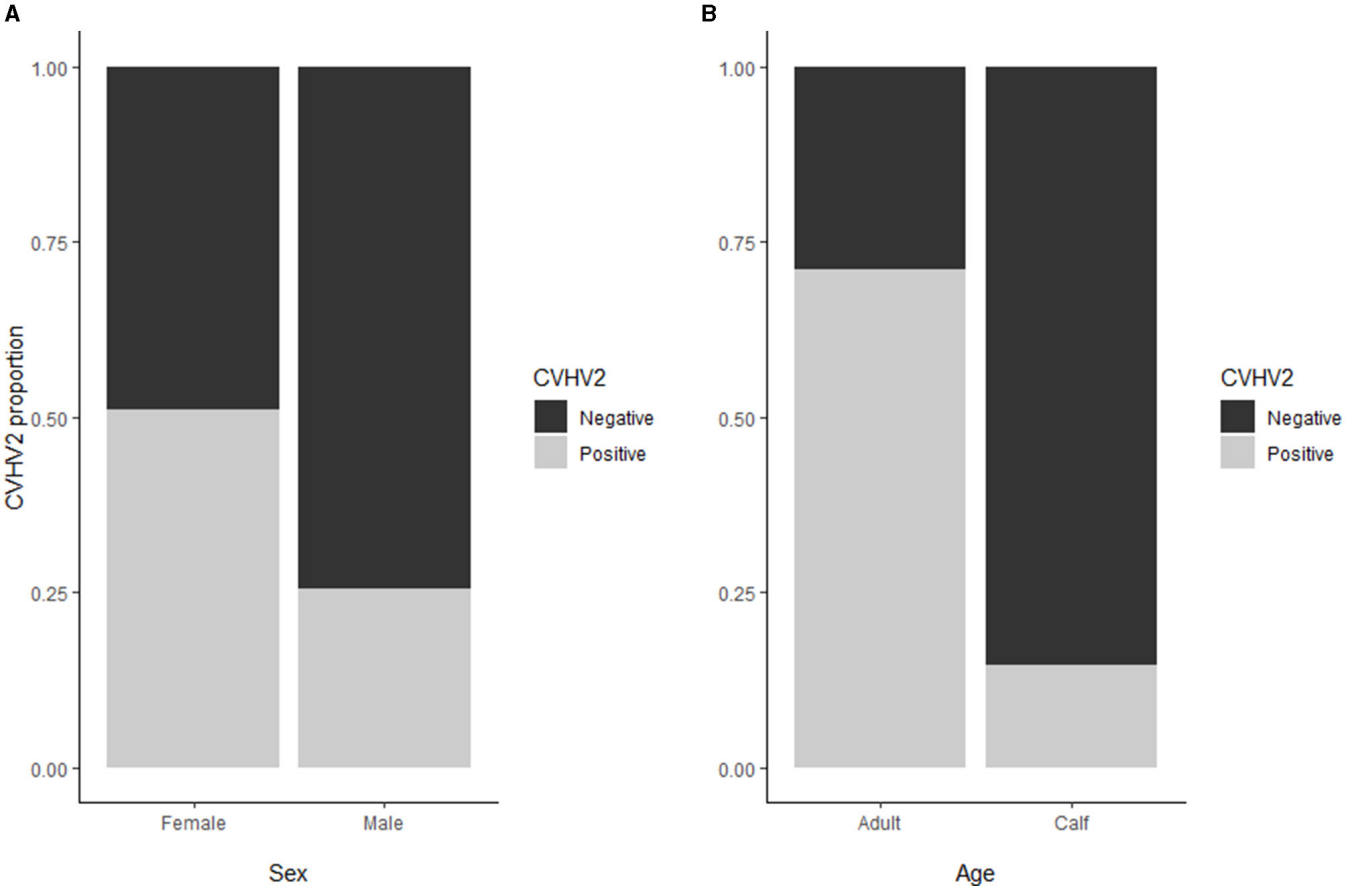
**(A)** For the alphaherpesvirus seroprevalence, no significant difference was found between males and females when correcting for age. **(B)** Alphaherpesvirus seroprevalence (overall 240/570; 42%) was higher in adult animals (71%) as compared to calves (15%).

Seroprevalence for alphaherpesvirus [overall 240/570; 42% (38;46)] was significantly higher in adult animals [197/277; 71% (66;76)] as compared to calves [43/293; 15% (11;19), *p* < 0.01], whereas no significant difference was found between males and females when correcting for age (Figure 3). There were no latitudinal or temporal trends in the alphaherpesvirus seroprevalence, although the three northernmost herds (i.e., Tana, Lakselv, and Tromsø) showed a high variability in seroprevalence, ranging from 21 to 63%.

**Figure 4:**
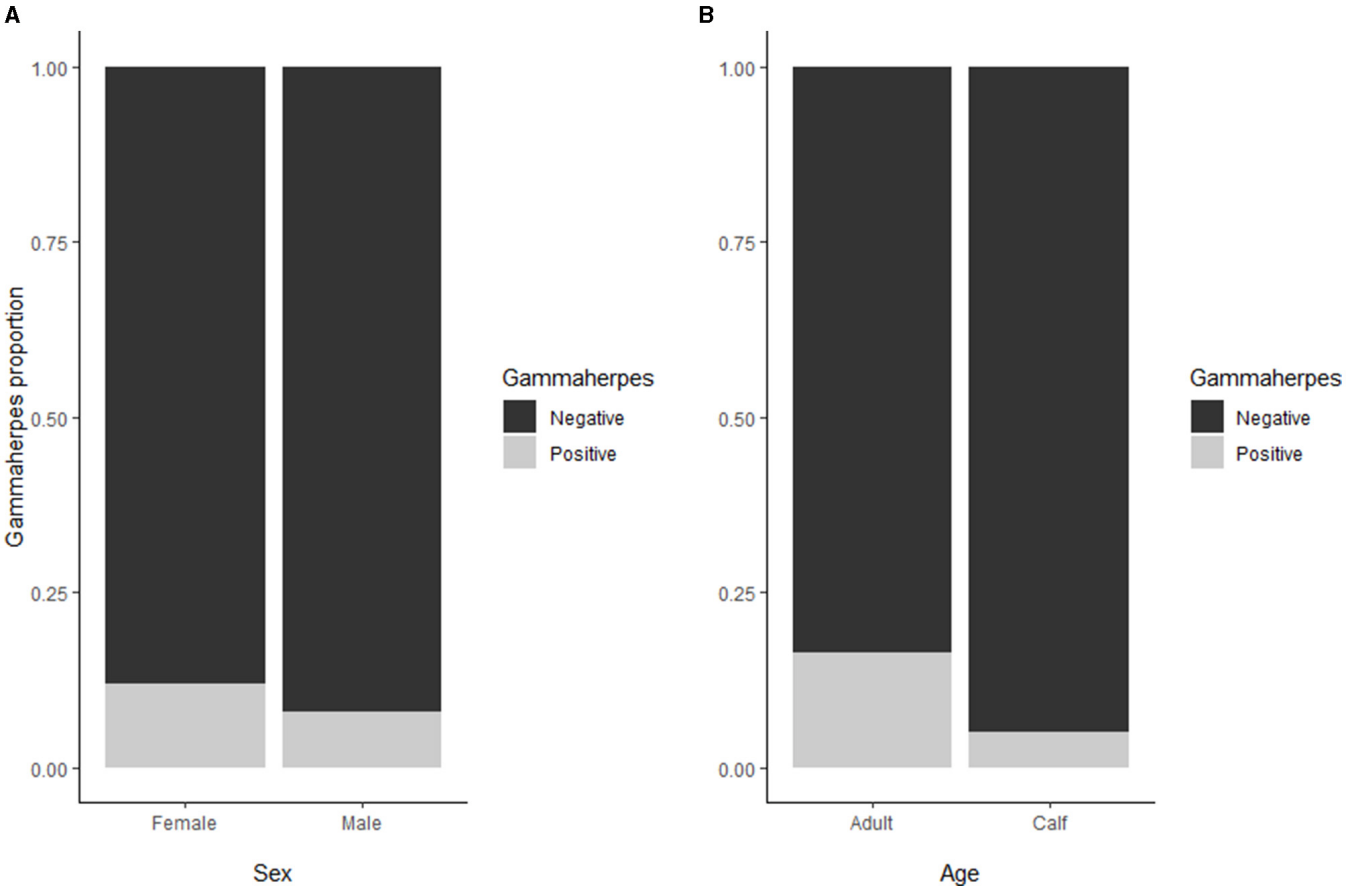
**(A)** For the MCFV seroprevalence, no significant difference was found between males and females. **(B)** The seroprevalence for MCFV (overall 58/550; 11% [95% C.I.: 8;13]) was higher in adults (16%) as compared to calves (5%).

Seroprevalence for MCFV [58/550; 11% (8;13)] was significantly higher in adults [44/270;16% (12;21)] as compared to calves [14/280; 5% (3,8), *p* < 0.01] whereas no significant difference was found between males and females (Figure 4). There was a significant increase in overall seroprevalence from ~10% during 2013–2016, to 22% in 2017 (*p* < 0.01). In addition, there was a trend with latitude, where three of the four northernmost herds (i.e., Tana, Lødingen, and Tromsø) showed a lower seroprevalence than their southern counterparts (*p* < 0.01).

**Figure 5:**
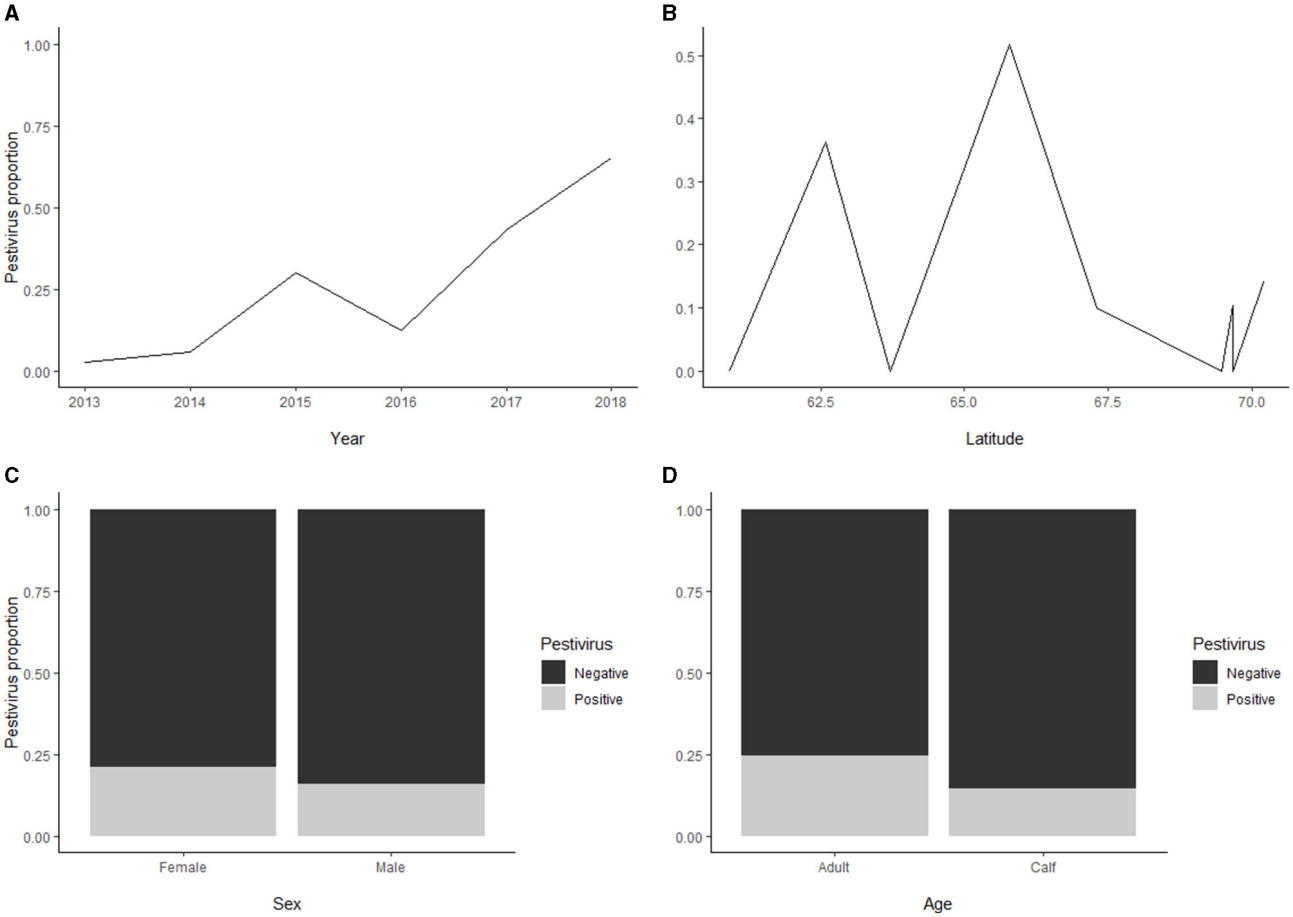
**(A)** For the pestivirus seroprevalence, there was an increasing temporal trend, from 4% in 2013 to 43% in 2017. **(B)** The pestivirus seroprevalence showed a high variability along the latitudinal gradient. **(C)** The seroprevalence for pestivirus revealed no significant difference between males and females. **(D)** The pestivirus seroprevalence (overall 110/570; 19%) was higher in adults (25%) as compared to calves (14%).

Seroprevalence for pestivirus [110/570; 19% (16;23)] was significantly higher in adults [68/277; 25% (20;30)] as compared to calves [42/293; 14% (11;19), *p* < 0.01] (Figure 5), whereas no significant difference was found between males and females. Furthermore, there was an increasing trend in overall seroprevalence with time, from 4% in 2013 to 43% in 2017, but with high variability along the latitudinal gradient.

None of the animals investigated had detectable antibodies against SBV or BTV.

PCR screening of nasal swabs for parapoxvirus revealed parapoxvirus-specific DNA of the *GIF* gene region in two nasal swab samples tested [2/486; 0.4% (0;1)], one from each of the two herds in Finnmark, both animals being female calves. PCR amplicons from one of these animals (Tana) were sequenced (consensus sequence, 296 nt, GenBank accession number MZ064575). A blast search (NCBI) indicated >99% sequence homology with a large number of ORFV isolates, including isolates from reindeer and caribou.

Thirteen reindeer, six from herd 5 (Hattfjelldal) and seven from herd 7 (Røros) had antibodies against alphaherpesvirus, MCFV and pestivirus at the same time, whereas parapoxvirus-specific DNA was not detected in any of these animals. Of the 13 animals, all except one were adults, 10 females, one male, and two with unrecorded sex.

## Discussion

This is the first thorough screening of viral infections in semi-domesticated reindeer in Norway, conducted over a period of five consecutive winter-seasons and representing the different geographical regions and varying ecosystems that are supporting reindeer herding in Norway.

Serum samples were of good quality, since they were collected either from live animals or from animals during the slaughter process and centrifuged for serum collection the same day. Further, the serological tests used have been validated and/or used for testing reindeer samples in previous studies (Table 2). Samples were obtained mostly during the main slaughter season of reindeer, after rut. Since the main slaughter season of bulls is prior to rut, the number of adult bulls in this study was restricted to 37, as compared to 198 females, which represents a gender bias in the sampling regime for this study.

The antibody prevalence against alphaherpesvirus, presumably CvHV2, was 42% (mean, all herds), varying from 21% in Tromsø to 63% in Tana. Similar figures have been published for semi-domesticated reindeer in Finnmark (49%; *n* = 3,062 animals) (7) as well as for seven populations of wild Eurasian tundra reindeer in southern Norway (29%; *n* = 831) (22). Together, these investigations demonstrate that an alphaherpesvirus is enzootic in all the reindeer populations in Norway, both wild and semi-domesticated.

Cervid herpesvirus 2 does presumably not cause disease outbreaks with high mortality, but its general distribution and high prevalence in most investigated reindeer herds makes its pathogenic potential interesting. It has been shown that CvHV2 can be the transmissible pathogen during outbreaks of infectious keratoconjunctivitis (IKC) in reindeer, paving the way for secondary bacterial infections (46), and that the virus alone is able to cause IKC under experimental conditions (14). The virus is also associated with respiratory infections and can be vertically transmitted to the reindeer fetus *in utero* (47, 48), and possibly contribute to abortion and reduced fitness of calves (11). Further, it has been demonstrated that the virus can experimentally be reactivated from latency, shed and isolated, after administration of glucocorticoids to seropositive animals (47, 49, 50). Thus, the impact of CvHV2 on the population level may be considerable and may be associated with stress and the reactivation of latent viruses.

The stress response in mammals includes activation of the hypothalamic-pituitary-adrenal axis, and the release of adrenal glucocorticoids. Experiments conducted in mice exposed to stress and subsequently inoculated with Herpes simplex virus 1 (HSV1) indicated that an exposure to stress, even prior to the infection, may compromise the individual's ability to mount an effective immune response, giving the pathogen time to replicate and spread (51). Studies in cattle with latent Bovine herpesvirus 1 (BHV1) infections and treated with glucocorticoids (i.e., mimicking stress) indicated that the reactivation process was more efficient compared to the mouse-HSV model, and that the glucocorticoid receptors stimulated viral replication upon reactivation (52).

These mechanisms have not been studied in detail for reindeer and CvHV2. However, it is likely that stimulation of the glucocorticoid receptors as a response to stress may reactivate latent CvHV2 infections. Further, stress would likely also make previously unexposed individuals (e.g., calves) more prone to develop disease and, with more severe symptoms, as in fact is commonly observed during outbreaks of infectious keratoconjunctivitis (46). The enzootic status of the virus documented here makes these stress-related mechanisms relevant for the whole reindeer herding region and for herding and management methods. This may be especially relevant when addressing potential effects of anthropogenic encroachments and fragmentation of reindeer pasture and climate change mitigation by increased feeding.

The seroprevalence against MCFV was 11%, ranging from 3% in Lødingen to 15% in Røros. For Lakselv (14%) in Finnmark and the three southernmost herds (Fosen 14%, Røros 15%, and Filefjell 13%), the seroprevalence was considerably higher than the 4% reported in Finnmark in a previous study [*n* = 3,339; (8)]. The disease MCF has been diagnosed in one semi-domesticated reindeer in Norway, displaying hair loss and thickening of the skin, with crusts on the muzzle, axillary regions and distal parts of the legs, and with presence of the bacterium *Trueperella pyogenes*. Both eyes displayed swollen eyelids and fibrinopurulent eye flood, as well as corneal opacity. Detection of Ovine herpesvirus 2 (OvHV2) specific DNA in the central nervous system (CNS) and nasal and conjunctival swabs by PCR verified the diagnosis (16). Pathological lesions in moose (*Alces alces*), roe deer (*Capreolus capreolus*), and red deer (*Cervus elaphus*), along with the detection of OvHV2 and Caprine herpesvirus 2 (CpHV2) specific DNA in the CNS of affected animals by PCR (53) demonstrated that also these cervid species are affected by MCF-viruses in Norway.

The overall seroprevalence against MCFV increased from ~10% for the period 2013–2016, to 23% in 2017. Although few animals constitute the baseline for these numbers, this may indicate the start of a trend that needs to be monitored in future studies. Whether reindeer in general are exposed to OvHV2, CpHV2 or other hitherto unidentified gammaherpesviruses that might be reindeer-associated remains to be proven. However, in a recent study, samples from semi-domesticated (*n* = 39) and wild (*n* = 35) Norwegian reindeer were tested using a panherpesvirus DNA polymerase (DPOL) PCR, revealing 11 and 17 PCR-positive reindeer, respectively. When testing the PCR-positive individuals further using primers targeting the glycoprotein B gene (gB), four semi-domesticated and 15 wild animals tested positive. Amplicon sequences from both genes indicated a high degree of sequence homology among the reindeer, corresponding to a novel gammaherpesvirus species tentatively named Rangiferine gammaherpesvirus 1, which may be a reindeer-specific gammaherpesvirus (10). The potential clinical impact of gammaherpesvirus infections especially with the viruses in MCFV group in reindeer remains to be elucidated.

The seroprevalence against pestivirus varied from 0% in three herds (Lakselv, Fosen, and Filefjell) to 52 and 37% in Hattfjelldal and Røros, respectively, with an overall mean of 19% (*n* = 570). This is slightly higher than what was found in a larger study conducted in Finnmark [13%, *n* = 3,339; (9)]. No pestivirus has been isolated from wild or semi-domesticated reindeer, but a pestivirus, designated V60, was isolated from a reindeer that suffered from diarrhea and anorexia in a German zoo in 1996 (54). Characterization of the isolate indicated that it was most closely related to Border disease virus type 2 (Pestivirus D; BDV-2) (55–57). An experimental inoculation of reindeer with BVDV caused loose stool with blood and mucus, serous mucopurulent nasal discharge and transient coronitis and laminitis (58).

It is unknown which type of pestivirus that has caused the immune response in this study. Thus, it is difficult to know which impact such infections may have on the reindeer populations. A transient infection in reindeer [e.g., BVDV; (58)] may be subclinical or cause only mild symptoms, whereas persistent infections, similar to mucosal disease (MD) in cattle and border disease (BD) in sheep, may cause severe symptoms. MD in cattle is characterized by high fever, salivation, anorexia and ulcers in the gastro-intestinal tract, whereas BD in small ruminants is characterized by abortion, stillbirths and the birth of small offspring, with abnormal body conformation, tremor and hairy fleece (“hairy shakers”) (59). This study has identified high seroprevalence in reindeer herds in Hattfjelldal and Røros. We have no obvious explanation for the significant increase in seroprevalence recorded for pestivirus during the sampling years. The recorded increase from 4% in 2013 to 43% in 2017 may reflect a real increase of exposure of the herds during this period or represent an arbitrary finding due to the opportunistic sampling regime. However, these findings could be followed up to further elucidate the role of pestivirus infections in semi-domesticated reindeer.

Interestingly, we detected parapoxvirus-specific DNA in one herd in western Finnmark and one in eastern Finnmark. Parapoxvirus-specific DNA has previously been detected in various tissues in reindeer carcasses from Finnmark with no clinical symptoms of contagious ecthyma (60), but the disease has never been reported in reindeer from this region. ORFV is presumably transmitted from small ruminants and the findings in this and previous studies may indicate that the virus is introduced to reindeer from time to time, maybe without causing clinical symptoms or that such symptoms are not recognized or reported. Lesions in the skin may not affect the health of the animal to a large extent, but when disseminated lesions appear in the oral mucosa it affects the ability of the animal to feed (26) and may cause substantial mortality during severe outbreaks (25).

None of the tested reindeer had antibodies against BTV and SBV. Climatic changes may affect the presence, distribution and biological activity of arthropods, including mosquitos, midges and ticks, in the reindeer herding regions, which may also alter the exposure of reindeer to BTV, SBV and other pathogens in the future. It is thus necessary to monitor reindeer for potential pathogens even though they are not known as being present and causing disease today. Another impact from climate change is increased rain-on-snow events and more frequent freeze-thaw cycles, creating ice-locked pastures for reindeer (61). One mitigating measure to avoid starvation is supplementary feeding. However, feeding may also represent increased stress and animal density and contribute to poor hygiene on feeding spots and in corrals. Virus infections (e.g., CvHV2, pestivirus, parapoxvirus, and others) may cause disease by themselves, but also facilitate secondary bacterial infections by causing lesions in skin and mucosal membranes. One such secondary bacterial infection is oral necrobaciullosis caused by *Fusobacterium necrophorum*, that may cause severe outbreaks in feeding corrals (16, 62, 63).

## Concluding Remarks

This study confirms that infections with alphaherpesvirus, gammaherpesvirus (MCFV group), and pestivirus are enzootic in semi-domesticated reindeer, also in herding regions south of Finnmark, whereas reindeer from none of the investigated geographical regions were exposed to BTV and SBV. The potential impact of alphaherpesvirus (i.e., CvHV2) infections has previously been demonstrated, with its role in the pathogenesis of IKC as the most prominent, whereas the role of MCFV and pestivirus infections in reindeer is less investigated. However, the high prevalence of exposure to these viruses, especially in some of the herds, may suggest that they may have an overall impact on reindeer health. It is in fact difficult to identify limited pathogenicity potentials, such as reduced reproductive success and poor fitness of calves, and how these infections may impact or facilitate other pathogens. This study also indicated that, even if disease outbreaks are not reported, parapoxvirus may be circulating among reindeer. Supplementary feeding of reindeer is rapidly adopted and is increasingly used by reindeer herders. Corralling and feeding may, however, may increase the stress load of the animals and lead to increased animal contact and poor hygienic conditions on feeding spots and in corrals, which may facilitate the transmission of pathogens and the severity of infectious diseases. Thus, the enzootic virus infections addressed in this study may become more important in the future management of reindeer health.

## Data Availability Statement

The datasets presented in this article are not readily available because the data on which the article is based on, contains personal data on identifiable reindeer herders and their animals. Requests to access the datasets should be directed to the corresponding author.

## Ethics Statement

Sampling was conducted in cooperation with the herders as a general health surveillance of the herds when animals were gathered and handled for other purposes, and the study was not classified as an animal experiment. Written informed consent was obtained from the owners for the participation of their animals in this study.

## Author Contributions

MT designed the study and drafted the manuscript. JSR, IHN, OCK, and MT conducted field work. EMB, CC, and HL conducted serological screenings. FJAM conducted the statistics. JSR, IHN, EMB, HL, CC, FJAM, and MT evaluated the results. All authors contributed to the manuscript and approved the final version.

## Funding

The study was funded by grants from the Norwegian Reindeer Development Fund (RUF; Climate and reindeer diseases) and by the Fram Center flagship Klimaeffekter på økosystemer, landskap, lokalsamfunn og urfolk, grant nr. 362256. The Open Access publication charges for this article have been funded by a grant from the publication fund of UiT The Arctic University of Norway.

## Conflict of Interest

The authors declare that the research was conducted in the absence of any commercial or financial relationships that could be construed as a potential conflict of interest.

## Publisher's Note

All claims expressed in this article are solely those of the authors and do not necessarily represent those of their affiliated organizations, or those of the publisher, the editors and the reviewers. Any product that may be evaluated in this article, or claim that may be made by its manufacturer, is not guaranteed or endorsed by the publisher.

## References

[1] Riseth JÅ Tømmervik H Forbes BC. Sustainable and resilient reindeer herding. In: Tryland M, Kutz S, editors, Reindeer and Caribou - Health and Disease. Boca Raton, FL: CRC Press - Taylor & Francis. (2019). p. 23–43.

[2] Eloranta EJ Timisjarvi M Nieminen J Leppaluoto O Vuolteenaho O. Hormonal changes in reindeer (*Rangifer tarandus tarandus*) during silent heat. In: Milne JA, editor, Recent Developments in Deer Biology. Aberdeen: Macaulay Land Use Research Institute. (1994). p. 151.

[3] Tryland M Nymo IH Sánchez Romano J Mørk T Klein J Rockström U. Infectious disease outbreak associated with supplementary feeding of semi-domesticated reindeer. Front Vet Sci. (2019) 6:126. 10.3389/fvets.2019.00126 31058176 PMC6482261

[4] Anonymous. Ressursregnskap for Reindriftsnæringen. Annual Reports 2014–2018. Alta: Norwegian Reindeer Husbandry Authority. (2019). Available online at: https://www.landbruksdirektoratet.no/nb/statistikk-og-utviklingstrekk/reindrift (accessed May 9, 2021).

[5] Stuen S Krogsrud J Hyllseth B Tyler NJC. Serosurvey of three virus infections in reindeer in northern Norway and Svalbard. Rangifer. (1993) 13:215–9. 10.7557/2.13.4.1120

[6] Tryland M Mørk T Ryeng KA Sørensen KK. Evidence of parapox-, alphaherpes- and pestivirus infections in carcasses of semi-domesticated reindeer (*Rangifer tarandus tarandus*) from Finnmark, Norway. Rangifer. (2005) 25:75–83. 10.7557/2.25.2.255

[7] das Neves CG Thiry J Skjerve E Yoccoz N Rimstad E Thiry E. Alphaherpesvirus infections in semidomesticated reindeer: a cross-sectional serological study. Vet Microbiol. (2009) 139:262–9. 10.1016/j.vetmic.2009.06.013 19604658

[8] das Neves CG Ihlebæk HM Skjerve E Hemmingsen W Li H Tryland M. Gammaherpesvirus infection in semidomesticated reindeer (*Rangifer tarandus tarandus*): a cross-sectional, serologic study in northern Norway. J Wildl Dis. (2013) 49:261–9. 10.7589/2012-07-185 23568901

[9] das Neves CG Wensman JJ Nymo IH Skjerve E Alenius S Tryland M. Pestivirus infections in semi-domesticated Eurasian tundra reindeer (*Rangifer tarandus tarandus*): a retrospective cross-sectional serological study in Finnmark County, Norway. Viruses. (2019) 12:29. 10.3390/v12010029 31888097 PMC7019806

[10] das Neves CG Sacristán C Madslien K Tryland M. Gammaherpesvirus in cervid species from Norway: characterization of a new virus in wild and semi-domesticated eurasian tundra reindeer (*Rangifer tarandus tarandus*). Viruses. (2020) 12:E876.32796534. 10.3390/v12080876 32796534 PMC7471987

[11] das Neves CG Roth S Rimstad E Thiry E Tryland M. Cervid herpesvirus 2 infection in reindeer: a review. Vet Microbiol. (2010) 143:70–80. 10.1016/j.vetmic.2010.02.015 20207086

[12] Evans AL das Neves CG Finstad GF Beckmen KB Skjerve E Nymo IH. Evidence of alphaherpesvirus infections in Alaskan caribou and reindeer. BMC Vet Res. (2012) 8:5. 10.1186/1746-6148-8-5 22243919 PMC3274481

[13] Carlsson AM Curry P Elkin B Russell D Veitch A Branigan M. Multi-pathogen serological survey of migratory caribou herds: a snapshot in time. PLoS ONE. (2019) 14:e0219838. 10.1371/journal.pone.0219838 31365561 PMC6668789

[14] Tryland M Sánchez Romano J Marcin N Nymo IH Josefsen TD Sørensen KK. Cervid herpesvirus 2 and not Moraxella bovoculi caused keratoconjunctivitis in experimentally inoculated semi-domesticated *Eurasian tundra* reindeer. Acta Vet Scand. (2017) 59:23. 10.1186/s13028-017-0291-2 28438213 PMC5404682

[15] Sánchez Romano J Mørk T Laaksonen S Ågren E Nymo IH Sunde M. Infectious keratoconjunctivitis in semi-domesticated *Eurasian tundra* reindeer (*Rangifer tarandus tarandus*): microbiological study of clinically affected and unaffected animals with special reference to cervid herpesvirus 2. BMC Vet Res. (2018) 14:15. 10.1186/s12917-018-1338-y 29338721 PMC5771138

[16] Tryland M das Neves CG Klein J Mørk T Hautaniemi M Wensman JJ. Virus infections and diseases. In: Tryland M, Kutz S, editors, Reindeer and Caribou - Health and Disease. Boca Raton, FL: CRC Press - Taylor & Francis. (2019). p. 273–303. 10.1201/9780429489617-8

[17] Russell GC Stewart JP Haig DM. Malignant catarrhal fever: a review. Vet J. (2009) 179:324–35. 10.1016/j.tvjl.2007.11.007 18760944

[18] Li H Cunha CW Taus NS Knowles DP. Malignant catarrhal fever: inching toward understanding. Annu Rev Anim Biosci. (2014) 2:209–33. 10.1146/annurev-animal-022513-114156 25384141

[19] Rehbinder C Belák S Nordkvist M. A serological, retrospective study in reindeer on five different viruses. Rangifer. (1991) 12:191–5. 10.7557/2.12.3.1044

[20] Kautto AH Alenius S Mossing T Becher P Belak S Larska M. Pestivirus and alphaherpesvirus infections in Swedish reindeer (*Rangifer tarandus tarandus* L.). Vet Microbiol. (2012) 156:64–71. 10.1016/j.vetmic.2011.10.018 22078277

[21] Omazic A Aurosell C Fedorov V Hagström Å Kantanen J Leijon M. Seroprevalence of pestivirus in Fennoscandian, Russian and Icelandic Eurasian tundra reindeer. Infect Ecol Epidemiol. (2019) 9:1682223, 10.1080/20008686.2019.1682223 31700582 PMC6830247

[22] Lillehaug A Vikøren T Larsen IL Akerstedt J Tharaldsen J Handeland K. Antibodies to ruminant alpha-herpesviruses and pestiviruses in Norwegian cervids. J Wildl Dis. (2003) 39:779–86. 10.7589/0090-3558-39.4.779 14733272

[23] Nordkvist M. Munvårtsjuka - en ny rensjukdom? Rennäringsnytt. (1973) 8–9:6–8.

[24] Kummeneje K Krogsrud J. Contagious ecthyma (orf) in reindeer (*Rangifer t. tarandus*). Vet Rec. (1979) 105:60–1. 10.1136/vr.105.3.60 576046

[25] Büttner M von Einem C McInnes C Oksanen A. Klinik und Diagnostik einer schweren Parapocken-Epidemie beim Rentier in Finland. Tierärztl Prax. (1995) 23:614–8. 8585082

[26] Tryland M Josefsen TD Oksanen A Aschfalk A. Contagious ecthyma in Norwegian semidomesticated reindeer (*Rangifer tarandus tarandus*). Vet Rec. (2001) 149:394–5. 10.1136/vr.149.13.394 11601519

[27] Damon I. Poxviruses. In: Knipe DM, Howley PM, editors, Fields Virology. London: Lippincott, Williams & Wilkins. (2007). p. 2947–75.

[28] Klein J Tryland M. Characterisation of parapoviruses isolated from Norwegian semi-domesticated reindeer (*Rangifer tarandus tarandus*). Virol J. (2005) 2:79. 10.1186/1743-422X-2-79 16143041 PMC1242257

[29] Ruiz-Fons F Sánchez-Matamoros A Gortázar C Sánchez-Vizcaáno JM. The role of wildlife in bluetongue virus maintenance in Europe: lessons learned after the natural infection in Spain. Virus Res. 182:50–58. 10.1016/j.virusres.2013.12.031 24394295

[30] Sánchez Romano J Grund L Obiegala A Nymo IH Ancin-Murguzur FJ Li H. A multi-pathogen screening of captive reindeer (*Rangifer tarandus*) in Germany based on serological and molecular assays. Front Vet Sci. (2019) 6:461. 10.3389/fvets.2019.00461 31921918 PMC6933772

[31] Gibbens N. Schmallenberg virus: a novel viral disease in northern Europe. Vet Rec. (2012) 170:58. 10.1136/vr.e292 22247205

[32] Wernike K Conraths F Zanella G Granzow H Gache K Schirrmeier H. Schmallenberg virus–two years of experiences. Prev Vet Med. (2014) 116:423–34. 10.1016/j.prevetmed.2014.03.021 24768435

[33] Wisløff H Nordvik BS Sviland S Tønnessen R. The first documented clinical case of Schmallenberg virus in Norway: fetal malformations in a calf. Vet Rec. (2014) 174:120. 10.1136/vr.102149 24399664

[34] das Neves CG Roger M Yoccoz NG Rimstad E Tryland M. Evaluation of three commercial bovine ELISA kits for detection of antibodies against alphaherpesviruses in reindeer (*Rangifer tarandus tarandus*). Acta Vet Scand. (2009) 51:9. 10.1186/1751-0147-51-9 19272136 PMC2663558

[35] das Neves CG Rimstad E Tryland M. Cervid herpesvirus 2 causes respiratory and fetal infections in semidomesticated reindeer. J Clin Microbiol. (2009) 47:1309–13. 10.1128/JCM.02416-08 19279181 PMC2681864

[36] Li H McGuire TC Muller-Doblies UU Crawford TB. A simpler, more sensitive competi- tive inhibition enzyme-linked immunosorbent assay for detection of antibody to malignant catarrhal fever viruses. J Vet Diagn Invest. (2001) 13:361–4. 10.1177/104063870101300417 11478614

[37] Deregt D Masri SA Cho HJ Bielefeldt OH. Monoclonal antibodies to the p80/125 gp53 proteins of bovine viral diarrhea virus: their potential use as diagnostic reagents. CanJVetRes. (1990) 54:343–8. 1696159 PMC1255667

[38] Vandenbussche F Vanbinst T Verhevden B Van Dessel W Demeestere L Houdart P. Evaluation of antibody-ELISA and real-time RT-PCR for the diagnosis and profiling of bluetongue virus serotype 8 during the epidemic in Belgium in 2006. Vet Microbiol. (2007) 129:15–27. 10.1016/j.vetmic.2007.10.029 18093753

[39] Linden A Desmecht D Volpe R Wirtgen M Pirson J Paternostre J. Epizootic Spreads of Schmallenberg virus among wild cervids, Belgium, fall 2011. Emerg Infect Dis. (2012) 18:2006–8. 10.3201/eid1812.121067 23171763 PMC3557893

[40] Li H Keller J Knowles DP Crawford TB. Recognition of another member of the malignant catarrhal fever virus group: an endemic gamma- herpesvirus in domestic goats. J Gen Virol. (2001) 82:227–32. 10.1099/0022-1317-82-1-227 11125175

[41] Tavernier P Sys SU De Clercq K De Leeuw I Caij AB De Baere M. Serologic screening for 13 infectious agents in roe deer (*Capreolus capreolus*) in Flanders. Infect Ecol Epidemiol. (2015) 5:29862. 10.3402/iee.v5.29862 26609692 PMC4660936

[42] Malmsten A Malmsten J Blomqvist G Näslund K Vernersson C Hägglund S. Serological testing of Schmallenberg virus in Swedish wild cervids from 2012 to 2016. BMC Vet Res. (2017) 13:84. 10.1186/s12917-017-1005-8 28376790 PMC5379663

[43] Inoshima Y Morooka A Sentsui H. Detection and diagnosis of parapoxvirus by the polymerase chain reaction. J Virol Methods. (2000) 84:201–8. 10.1016/S0166-0934(99)00144-5 10680970

[44] Deane D McInnes C Percival A Wood A Thomson J Lear A. Orf virus encodes a novel secreted protein inhibitor of granulocyte-macrophage col- ony-stimulating factor and interleukin-2. J Virol. (2000) 74:1313–20. 10.1128/JVI.74.3.1313-1320.2000 10627542 PMC111466

[45] R Core Team. R: A Language and Environment for Statistical Computing. Vienna: R Foundation for Statistical Computing (2021). Available online at: https://www.R-project.org/ (accessed August 1, 2021).

[46] Tryland M das Neves CG Sunde M Mørk T. Cervid herpesvirus 2, the primary agent in an outbreak of infectious keratoconjunctivitis in semidomesticated reindeer. J Clin Microbiol. (2009) 47:3707–13. 10.1128/JCM.01198-09 19726598 PMC2772613

[47] das Neves CG Mørk T Thiry J Godfroid J Rimstad E Thiry E. Cervid herpesvirus 2 experimentally reactivated in reindeer can produce generalized viremia and abortion. Virus Res. (2009) 145:321–8. 10.1016/j.virusres.2009.08.002 19699769

[48] das Neves CG Mørk T Godfroid J Sørensen KK Breines E Hareide E. Experimental infection of reindeer with cervid herpesvirus 2. Clin Vaccine Immunol. (2009) 16:1758–65. 10.1128/CVI.00218-09 19846680 PMC2786388

[49] Ek-Kommonen C Pelkonen S Nettleton PF. Isolation of a herpesvirus serologically related to bovine herpesvirus 1 from a reindeer (*Rangifer tarandus*). Acta Vet Scand. (1986) 27:299–301. 10.1186/BF03548174 3026156 PMC8189397

[50] Rockborn G Rehbinder C Klingeborn B Lefler M Klintevall K Nikkilä T. The demonstration of a herpesvirus, relatedto bovine herpesvirus 1, in reindeer with ulcerative and necrotizing lesions ofthe upper alimentary tract and nose. Rangifer. (1990) 3:373–84. 10.7557/2.10.3.882

[51] Elftman MD Hunzeker JT Mellinger JC Bonneau RH Norbury CC Truckenmiller ME. Stress-induced glucocorticoids at the earliest stages of herpes simplex virus-1 infection suppress subsequent antiviral immunity, implicating impaired dendritic cell function. J Immunol. (2010) 184:1867–75. 10.4049/jimmunol.0902469 20089700 PMC3701455

[52] Kook I Doster A Jones C. Bovine herpesvirus 1 regulatory proteins are detected in trigeminal ganglionic neurons during the early stages of stress-induced escape from latency. J Neurovirol. (2005) 21:585–91. 10.1007/s13365-015-0339-x 25860382

[53] Vikøren T Li H Lillehaug A Jonassen CM Böckerman I Handeland K. Malignant catarrhal fever in free-ranging cervids associated with OvHV-2 and CpHV-2 DNA. J Wildl Dis. (2006) 42:797–807. 10.7589/0090-3558-42.4.797 17255446

[54] Becher P Orlich M Kosmidou A Konig M Baroth M Thiel HJ. Genetic diversity of pestiviruses: identification of novel groups and implications for classification. Virology. (1999) 262:64–71. 10.1006/viro.1999.9872 10489341

[55] Avalos-Ramirez R Orlich M Thiel HJ Becher P. Evidence for the presence of two novel pestivirus species. Virology. (2001) 286:456–65. 10.1006/viro.2001.1001 11485413

[56] Becher P Ramirez R Avalos MO Rosales SC Konig M Schweizer M. Genetic and antigenic characterization of novel pestivirus genotypes: implications for classification. Virology. (2003) 311:96–104. 10.1016/S0042-6822(03)00192-2 12832207

[57] Giangaspero M Harasawa R Muschko K Buttner M. Characteristics of the 5' untranslated region of wisent (*Bison bonasus*) and reindeer (*Rangifer tarandus*) Pestivirus isolates. Vet Ital. (2006) 42:165–72. 20429058

[58] Morton J Evermann JF Dieterich RA. Experimental infection of reindeer with bovine viral diarrhea virus. Rangifer. (1990) 10:75–7. 10.7557/2.10.2.797

[59] Radostits OM Gay CC Blood DC Hindchliff KW. Diseases caused by viruses and chlamydia I. Bovine virus diarrhea, mucosal disease, bovine pestivirus disease complex. In: Radostits OM, Gay C, Hinchcliff KW, Constable PD, editors. Veterinary Medicine. A Textbook of the Diseases of Cattle, Sheep, Pigs, Goats and Horses. Philadelphia, PA: Saunders Ltd. (2000). p. 1085–105.

[60] Tryland M. Asymptomatic parapoxvirus infections in semi-domesticated reindeer (*Rangifer tarandus tarandus*), In: Proceedings of the XIVth International Poxvirus & Iridovirus Workshop; 2002 Sep 20–25. New York (2002). p. 169.

[61] Mallory CD Boyce MS. Observed and predicted effects of climate change on Arctic caribou and reindeer. Environ Ver. (2017) 26:13–25. 10.1139/er-2017-0032

[62] Josefsen TD Mørk T Nymo IH. Bacterial infections and diseases. In Tryland M, Kutz S, editors, Reindeer and Caribou. Health and Disease. CRC Press: Boca Raton, FL (2019). p. 237–71. 10.1201/9780429489617-7

[63] Laaksonen S. Assessment and treatment of reindeer diseases. In Tryland M, Kutz S, editors, Reindeer and Caribou - Health and Disease. CRC Press; Taylor & Francis: Boca Raton, FL (2019). p. 383–444. 10.1201/9780429489617-12

